# Emergence of a Multidrug-Resistant *Enterobacter hormaechei* Clinical Isolate from Egypt Co-Harboring *mcr-9* and *bla*_VIM-4_

**DOI:** 10.3390/microorganisms8040595

**Published:** 2020-04-20

**Authors:** Ahmed M. Soliman, Fumito Maruyama, Hoda O. Zarad, Atsushi Ota, Hirofumi Nariya, Toshi Shimamoto, Tadashi Shimamoto

**Affiliations:** 1 Laboratory of Food Microbiology and Hygiene, Graduate School of Biosphere Science, Hiroshima University, Higashi-Hiroshima 739-8528, Japan; ahmed_soliman@pharm.kfs.edu.eg (A.M.S.); hodaothmanzarad@gmail.com (H.O.Z.); 2 Department of Microbiology and Immunology, Faculty of Pharmacy, Kafrelsheikh University, Kafr El-Sheikh 33516, Egypt; 3 Microbial Genomics and Ecology, Office of Academic Research and Industry-Government Collaboration, Hiroshima University, Higashi-Hiroshima 739-8530, Japan; fumito@hiroshima-u.ac.jp (F.M.); level997@gmail.com (A.O.); 4 Laboratory of Food Microbiology and Hygiene, Graduate School of Integrated Sciences for Life, Hiroshima University, Higashi-Hiroshima 739-8528, Japan; nariya@hiroshima-u.ac.jp (H.N.); tsima@hiroshima-u.ac.jp (T.S.)

**Keywords:** *mcr-9*, Egypt, VIM-4, IncHI2, WGS, *Enterobacter hormaechei*

## Abstract

This study describes the first full genomic sequence of an *mcr-9* and *bla*_VIM-4_-carrying multidrug-resistant *Enterobacter hormaechei* clinical isolate from Egypt. The strain was isolated in April 2015 from the sputum of a patient in Cairo, Egypt. The *mcr-9* and *bla*_VIM-4_ genes were identified by PCR screening and DNA sequencing; the isolate was subjected to antimicrobial susceptibility testing, conjugation experiments, and whole genomic sequencing. *mcr-9* and *bla*_VIM-4_ were carried by an IncHI2 plasmid, pAMS-38a (281,121 bp in size); the plasmid also carried genes conferring resistance against sulfonamides (*sul1*), quinolones (*qnrA1*), trimethoprim (*dfrA1*), β-lactams (*bla*_TEM-1B_), aminoglycosides (*aac (6’)-II*, *aadA23*, *aadA2b*, and *ant(2’’)-Ia*). The strain was susceptible to colistin (MIC, <0.25 μg/mL); this could be due to the absence of the *qseC*/*qseB* regulatory system located downstream of *mcr-9* in Enterobacterales, which is involved in the induction of colistin-resistance. The genetic context of *mcr-9* and *bla*_VIM-4_ was identified as IS*1*-*mcr-9*-IS*903*-*pcoS-∆pcoE-rcnA* and *intI1*-*bla*_VIM-4—_*aac (6’)-II*-*dfrA1*-*∆aadA23*-*smr*-IS*Pa21*-*qacE∆1*, respectively. This is the first report of an *mcr-9* and *bla*_VIM-4_ /IncHI2-carrying multidrug-resistant *E. hormaechei* clinical isolate from Africa and the Middle East. Plasmids of the IncHI2 group and the two insertion sequences (IS*1*, and IS*903*) might be the main vehicles for dissemination of *mcr-9*. Further screening for *mcr-9* is essential for identifying its incidence and to prevent its dissemination.

## 1. Introduction

Problems associated with the development and spread of antimicrobial resistance (AMR) in clinical practice are increasing and are currently being viewed as a major threat to public health globally [1,2]. Carbapenem resistance in Enterobacterales is mediated through the expression of a large set of carbapenemase-encoding genes: particularly, Ambler class A β-lactamases (KPC, FRI, GES, SME), Ambler class B metallo-β-lactamases (VIM, NDM, IMP, SIM, GIM, SPM), and Ambler class D enzymes (OXA-type) [3,4]. Verona integron-mediated metallo-β-lactamase (VIM) is located as a gene cassette in a class 1 integron with genes for resistance against trimethoprim or aminoglycosides [5]. The *bla*_VIM-4_ gene encodes resistance against all β-lactams except aztreonam and was previously reported in *Pseudomonas aeruginosa*, *Enterobacter cloacae*, or other Enterobacterales from Egypt, Kuwait, and United Arab Emirates [5,6,7,8]. Recently, we reported *bla*_VIM-2_ and *bla*_VIM-24_ from clinical *P. aeruginosa* strains from Egypt [5].

Colistin is one of the last-resort antibiotics for treating infections caused by carbapenem resistant Gram-negative bacteria. The activity and efficacy of colistin has been challenged by the global spread of the self-transmissible mobilized colistin-resistance genes (*mcr*). In 2016, the first *mcr-1* gene was reported from *Escherichia coli* and *Klebsiella pneumoniae* isolated from patients, food, and animals in China [9]. MCR-1 acts by modifying the lipid A part of the lipopolysaccharide in Gram-negative bacteria by adding phosphoethanolamine, which reduces the binding affinity to colistin [9]. As of 7 January 2020, 10 variants of the *mcr* genes are available in the Bacterial Antimicrobial Resistance Reference Gene Database (https://www.ncbi.nlm.nih.gov/pathogens/isolates#/refgene/); however, only *mcr-1* has been previously reported from Egypt [10], and little is yet known about the prevalence of other *mcr* variants in Egypt.

Here, for the first time, we describe the complete genomic sequence of an *E. hormaechei* clinical isolate carrying the *mcr-9* and *bla*_VIM-4_ genes on an IncHI2 plasmid from Africa and the Middle East generated using the Illumina MiniSeq and Oxford Nanopore sequencing platforms.

## 2. Materials and Methods

### 2.1. Bacterial Isolation and Identification

The *E. hormaechei* strain AMS-38 was isolated from the sputum swab of a patient in Cairo, Egypt in April 2015. The strain was identified by 16S ribosomal RNA gene sequencing using primers 27F and 1492R [5]. PCR and DNA sequencing were conducted to identify extended-spectrum β-lactamases, carbapenemase-encoding genes, 16S rRNA methylases, plasmid-mediated quinolone-resistance genes, and plasmid-mediated colistin-resistance genes (*mcr-1–mcr-8*) as previously described [5]. Additionally, *mcr-9* was detected by primers *mcr-9*-forward: 5’-CGGTACCGCTACCGCAATAT-3’ and *mcr-9*-reverse: 5’-ATAACAGCGAGACACCGGTT-3’ [11].

### 2.2. Antimicrobial Susceptibility Testing (AST)

A standardized broth microdilution (BMD) technique was used to determine the minimum inhibitory concentration (MIC) of various antimicrobial agents according to the standards and interpretive criteria described by the Clinical and Laboratory Standards Institute (CLSI, document M100-S24) and European Committee on Antimicrobial Susceptibility Testing (EU-CAST) (for colistin and tigecycline breakpoints) (http://www.eucast.org). For all experiments, the purified powder of each antibiotic was diluted following CLSI recommendations. *E. coli* ATCC 25922 was used as a control.

### 2.3. Filter-Mating Conjugation 

A Conjugation experiment was performed using the AMS-38 strain and the azide resistant *E. coli* J53 strain as the donor and recipient, respectively. Due to the thermo-susceptibility of IncHI2 plasmids (i.e., conjugation is impaired at 37 ˚C), we performed this experiment at 25–30 ˚C [12]. The transconjugants were selected on LB agar plates containing 100 μg/mL ampicillin and 100 μg/mL sodium azide. Colony-direct PCR was performed using VIM-F and VIM-R [5] and *mcr-9*-forward and *mcr-9*-reverse [11] primers to confirm the transfer of the plasmid carrying *mcr-9* and *bla*_VIM-4_.

### 2.4. Whole Genome Sequencing (WGS) and Analysis

An overnight bacterial culture of the AMS-38 strain was used to extract the total genomic DNA (gDNA) using a standard proteinase K method. The quality of the isolated gDNA was assessed using a Microdrop (Thermo Scientific™, Multiskan™ Sky Microplate Spectrophotometer, Waltham, MA, USA), Bioanalyzer (Agilent Technologies, Santa Clara, CA, USA), and a dsDNA fluorescent dye method using DeNovix (Wilmington, DE, USA). The sequencing library was constructed using a Nextera DNA Flex Library Prep Kit (Illumina, San Diego, CA, USA.) for Illumina MiniSeq. The DNA library for Oxford Nanopore sequencing was prepared using 400 ng of gDNA according to the Rapid Barcoding Sequencing kit (SQK-RBK004) (Oxford Nanopore Technologies, Oxford, UK). The sequencing libraries were purified using a Promega Size-Selective Purification System (Promega, Madison, WI, USA), and 11 μL of the sequencing library was loaded onto a FLO-MIN106 flow cell and sequenced with the GridION device (Oxford Nanopore Technologies) for 48 h. Hybrid assembly of both Illumina and Nanopore reads was performed using Flye v2.6 (https://github.com/fenderglass/Flye). Additionally, Pilon-v1.23 [13] was used to improve the genome assembly. The assembly and annotation of pAMS-38a were performed using DFAST [14]. The identification of AMS-38 was done with JSpeciesWS (http://jspecies.ribohost.com/jspeciesws/#analyse) using Tetra Correlation Search (TCS) against reference database Genome DB and the pairwise comparison using Average Nucleotide Identity calculation based on BLAST+ (ANIb). Antimicrobial resistance genes were identified by ResFinder-3.2 available at Center for Genomic Epidemiology (https://cge.cbs.dtu.dk/services/ResFinder/). The plasmid Inc groups, pMLST and multilocus sequence typing (MLST) were identified by PlasmidFinder (https://cge.cbs.dtu.dk/services/PlasmidFinder/), (https://cge.cbs.dtu.dk/services/pMLST/) and MLST 2.0 software (https://cge.cbs.dtu.dk/services/MLST/), respectively. The plasmids that were highly similar to pAMS-38a were detected by a Mash (v2.1.1) [15] distance search against the PLSDB v. 2019_10_07 [16]. The BRIG tool was used to perform a circular comparison between the complete sequence of pAMS-38 and the highly similar plasmids detected by a Mash distance search [17]. EasyFig tool was used to visualize and compare the genetic environment of *mcr-9* in its harboring plasmids (http://mjsull.github.io/Easyfig/).

### 2.5. Nucleotide Sequence Accession Numbers

The complete genome sequence of AMS-38 and the plasmids pAMS-38a, pAMS-38b, pAMS-38c, pAMS-38d were submitted to DDBJ/ENA/GenBank under BioProject ID: PRJNA616711.

## 3. Results and Discussion

### 3.1. AST of E. hormaechei AMS-38, and Conjugation of mcr-9 and bla_VIM-4_-Carrying Plasmid

The sequencing of the 16S ribosomal RNA gene identified a strain belonging to the *E. cloacae* complex. BMD showed that the strain AMS-38 was resistant to aztreonam (MIC, 32 μg/mL), ceftazidime (MIC, >256 μg/mL), ceftriaxone (MIC, >256 μg/mL), meropenem (MIC, 64 μg/mL), ertapenem (MIC, >64 μg/mL), kanamycin (MIC, 64 μg/mL), ciprofloxacin (MIC, 2 μg/mL), fosfomycin (MIC, 128 μg/mL), tetracycline (MIC, 16 μg/mL), and tigecycline (MIC, 4 μg/mL). The strain showed intermediate resistance to chloramphenicol (MIC, 16 μg/mL) and sensitivity to colistin (MIC, < 0.25 μg/mL). PCR screening and DNA sequencing identified the presence of the metallo-β-lactamase genes, *bla*_VIM-4_, *bla*_TEM-1_, AmpC β-lactamase, the newly described inducible colistin-resistance gene *mcr-9*, the fosfomycin glutathione-S-transferase gene *fosA*, and the plasmid-mediated quinolone-resistance gene *qnrA1*. The results showed that the *mcr-9* and *bla*_VIM-4_-carrying plasmid was successfully transferred to *E. coli* J53. The intact conjugative transfer locus in pAMS-38a was similar to that locus in plasmid pME-1a (accession no. CP041734), and found in two distinct regions, Tra1 and Tra2 illustrating the potential for horizontal gene transfer to other Gram-negative bacteria.

### 3.2. Characterization of the Genome of E. hormaechei AMS-38

By combining the short reads obtained from Illumina MiniSeq and the long reads obtained from Oxford Nanopore sequencing, we obtained assemblies with high-quality and sufficient for closing the genome and the plasmids contained in the strain. The chromosome of the AMS-38 strain was 4,914,941 bp in size with an average G + C content of 54.9%. Analyzing the average nucleotide identity (ANI) of the AMS-38 genome showed that the strain belonged to *E. hormaechei* subsp. *steigerwaltii* group (99.69% identity (ID) with 97.71% query coverage(QC) to *E. hormaechei* subsp. *steigerwaltii* (GCA 001011725) GN02001 followed by *Enterobacter* sp. MGH86 (99.62% ID with 95.21% QC), followed by *E. hormaechei* subsp. *steigerwaltii* 44524 (98.36% ID with 86.91% QC), followed by *E. hormaechei* subsp. *steigerwaltii* 44517 (98.36% ID with 86.92 QC)). Analysis of MLST of the strain AMS-38 showed that it belonged to ST133 (allelic profile 11-4-4-13-39-4-9).

### 3.3. Plasmids and Resistome Analysis of the Strain E. hormaechei AMS-38

The isolate contained four plasmids (pAMS-38a, pAMS-38b, pAMS-38c, pAMS-38d) ranging in size from 2,511 bp to 281,121 bp (Table 1). ResFinder detected that the intrinsic *bla*_ACT-7_ and *fosA* genes were located on the chromosome. Additionally, plasmid pAMS-38a contained 10 antimicrobial resistance genes encoding resistance against colistin (*mcr-9*), sulphonamide (*sul1*), quinolones (*qnrA1*), trimethoprim (*dfrA1*), β-lactam (*bla*_TEM-1B_, and *bla*_VIM-4_), and aminoglycosides (*aac (6’)-II*, *aadA23*, *aadA2b*, and *ant(2’’)-Ia*). The remaining three plasmids did not carry any resistance genes.

**Figure 1:**
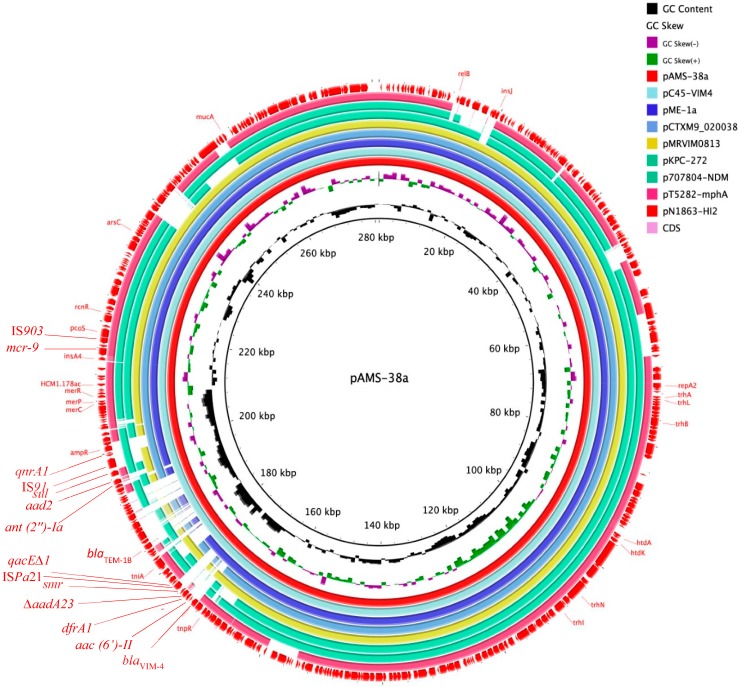
Plasmid structure of *mcr-9*/IncHI2 plasmids. The whole sequence of pAMS-38a was used as the reference. Genes and open reading frames (ORFs) are shown as arrows with their transcriptional orientations indicated by arrowheads. This figure was generated using the BRIG tool [17], and the plasmids were included in the following order: pAMS-38a (identified in this study), pC45-VIM4 (LT991958), pME-1a (CP041734), pCTXM9_020038 (CP031724), pMRVIM0813 (KP975077), pKPC-272 (CP008825), p707804-NDM (MH909331), pT5282-mphA (KY270852), and pN1863-HI2 (MF344583).

Plasmid pAMS-38a is an IncHI2 with Double Locus Sequence Type DLST1 of 281,121 bp in size encoding 361 predicted genes with an average G + C content of 46.8% (Figure 1 and Table 1). Plasmids of IncHI type are large in size (>200 kb) [12]. These plasmids have broad host range and have been commonly detected from several Enterobacterales species of clinical and environmental origin, such as *Salmonella enterica* serovars Typhi and Paratyphi A, *E. coli*, *K. pneumoniae*, and *E. cloacae* [12]. IncHI2 plasmids are characterized by carrying genes encoding heavy metal and antimicrobial resistance [12].

A Mash distance search against the PLSDB using the whole pAMS-38a sequence identified that it has high similarity to other IncHI2 plasmids (Figure 1 and Table 2), e.g., pC45-VIM4 (accession no. LT991958), pME-1a (accession no. CP041734) [18], pCTXM9_020038 (accession no. CP031724), pMRVIM0813 (accession no. KP975077), pKPC-272 (accession no. CP008825), p707804-NDM (accession no. MH909331), pT5282-mphA (accession no. KY270852), and pN1863-HI2 (accession no. MF344583). Alarmingly, all these plasmids carried the *mcr-9* gene with or without carbapenemase-encoding genes (i.e., *bla*_VIM_, *bla*_KPC_, and *bla*_NDM_) between 2012 to 2018, highlighting the early silent emergence and dissemination of *mcr-9.* A recent study conducted in the USA identified a similar *mcr-9* and *bla*_VIM-4_/IncHI2 plasmid pME-1a (accession no. CP041734) from an *E. hormaechei* strain isolated from a pediatric patient with a recent history of travel to Egypt [18]. Therefore, our study raises the concern of implementing antimicrobial surveillance plans and infection control policies for *mcr-9* to identify its incidence and to prevent its dissemination.

**Figure 2:**
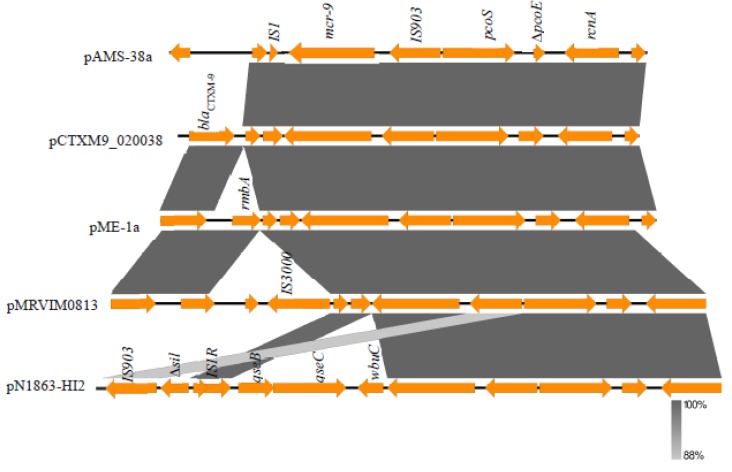
Comparison of the genetic environment of *mcr-9* gene in pAMS-38a and other closely related IncHI2 plasmids. The figure was drawn using the EasyFig tool. Two different genetic environments have been detected. IS*1*-*mcr-9*-IS*903* was detected from pAMS-38a (identified in this study), pME-1a (CP041734), pCTXM9_020038 (CP031724), pMRVIM0813 (KP975077)*,* and *qseB*-*qseC*-*wbuC*-*mcr-9*-IS*903* was detected from pN1863-HI2 (MF344583).

Analysis of the genetic environment of *mcr-9* revealed that it was surrounded upstream by IS*903* and downstream by IS*1.* Additionally, these two insertion sequences might be involved in the mobilization of *mcr-9*. This genetic environment has been reported previously from several IncHI2 plasmids (Figure 2) e.g., pME-1a (accession no. CP041734) [18], pCTXM9_020038 (accession no. CP031724), and pMRVIM0813 (accession no. KP975077). A different genetic organization, *qseB*-*qseC*-*wbuC*-*mcr-9*-IS*903*, has been detected from other IncHI2 plasmids e.g., pN1863-HI2 (accession no. MF344583), pT5282-mphA (accession no. KY270852), pMRVIM0813 (accession no. KP975077), and the sequenced plasmid contig from *E. coli* strain E68 from France [11]. In that study, Kieffer et al. showed that *mcr-9* is an acquired colistin-resistance gene induced by the action of the downstream two-component regulatory system encoded by *qseB*/*qseC* using subinhibitory concentrations of colistin [11]. Another study was conducted on a clinical *E. hormaechei* isolate from China with the coproduction of MCR-9 and NDM-1 [19]. The investigators identified *mcr-9* to be located on an IncHI2 plasmid with the downstream genes *wbuC* (for a cupin fold metalloprotein) and the *qseB*/*qseC* leading to colistin-resistance with an MIC of 16 μg/mL [19]. In our study, the sensitivity of the strain AMS-38 to colistin (MIC, <0.25 μg/mL) was probably due to the absence of *qseB*/*qseC.* It has been noted that *mcr-9* might have originated from *Buttiaxella* spp. due to the presence of a gene homologous to *wbuC* downstream of the chromosomal *mcr-9*-like gene in this genus [11,19]. Of note, *mcr-9* was first identified, during a routine in silico analysis, in a multidrug- resistant colistin-sensitive *S. enterica* serovar Typhimurium clinical isolate from the USA in 2010 [20]. Recently, *mcr-9* was detected among SHV-12-producing Enterobacterales from Swedish horses with a link to IncHI2 and IncHI2A plasmids [21]. Additionally, an extensively drug-resistant *bla*_NDM-1_-carrying *Klebsiella quasipneumoniae* was detected to harbor *mcr-9* on an IncHI2 plasmid for the first time in Latin America [22]. A novel mobile colistin resistance gene, *mcr-10*, was recently reported from a clinical *Enterobacter roggenkampii* isolate in China [23]. MCR-10 has 82.93% amino acids identity to MCR-9 [23]. Gene *mcr-10* was detected to be uninducible and increases the colistin MIC by 4 folds when cloned into a colistin-susceptible *E. roggenkampii* strain [23].

Based on the available literature, the frequency of detecting *mcr-9*-carrying Enterobacterales with susceptibility to polymyxins is high [18,20,21,22]. Additionally, we speculate a silent dissemination of *mcr-9* since its possession confers a decreased susceptibility, not resistance, to colistin as detected in several Enterobacterales [18,20,21,22]. On the other hand, only two studies detected *mcr-9* conferring clinical resistance to colistin [11,19]. Moreover, the expression of *mcr-9* was shown to be inducible due to the presence of the downstream *qseC*/*qseB* regulatory system [11]. Furthermore, the lack of these two genes downstream of the *mcr-9*-like gene in its original progenitor, *B. gaviniae*, suggests that their acquisition likely has been through an independent mobilization event with respect to the acquisition of *mcr-9* [11]. Similarly, that event might happen in *mcr-9*-carrying and colistin susceptible strains leading to the induction and colistin resistance. Until now, the clinical impact of *mcr-9*, in the colistin-susceptible strains lacking *qseC*/*qseB* system, is unknown. 

The class B1 metallo-β-lactamase *bla*_VIM-4_ was located on an In*416* class 1 integron. A BLASTn search using the whole *bla*_VIM-4_ integron sequence of pAMS-38a identified identical integrons from pC45-VIM4 (accession no. LT991958), pME-1a [18], pC309-VIM4 (accession number LT991955.1) (100% query coverage and 99.97% sequence identity). *bla*_VIM-4_ has been previously reported from *E. cloacae* and *P. aeruginosa* from Egypt, Kuwait, and United Arab Emirates [6,7,8,24]. The integron structure was *bla*_VIM-4_-*aacA7*-*dfrA1*-*ΔaadA23*-*smr*-IS*Pa21.* Another class 1 integron was detected in pAMS-38a with the following genetic structure: *intI1*-*ant (2’’)-Ia*-*aad2b-sul1*-IS*91*-*qnrA1*. A BLASTn analysis identified a similar integron from pCTXM9_020038 (accession no. CP031724; 100% query coverage and 99.88% sequence identity), and pC45-VIM4 (accession no. LT991958; 100% query coverage and 99.87% sequence identity). 

## 4. Conclusions

In conclusion, we reported the first complete genomic sequence of an *mcr-9*- and *bla*_VIM-4_-co-harboring *E. hormaechei* strain from Africa and the Middle East. Comparative genomics was used to assess the genetic environment of the *mcr-9* gene. Plasmids of the IncHI2 group and the two insertion sequences (IS*1*, and IS*903*) might be the main vehicles for dissemination of *mcr-9*. Screening for *mcr-9* and other *mcr* genes is essential in Egypt to determine its prevalence and to prevent its spread.

## Figures and Tables

**Table 1:** Features of chromosome and the plasmids identified in *E. hormaechei* AMS-38 from Egypt.

Sample	Size (bp)	GC%	No. of CDSs	MLST or pMLST	Inc Type *	AMR Gene
Chromosome	4,914,941	54.9	6279	ST133	ND	*fosA*, *bla*_ACT-7_
pAMS-38a	281,121	46.8	361	ST1	IncHI2	*mcr-9*, *sul1*, *qnrA1*, *dfrA1*, *bla*_TEM-1B_, *bla*_VIM-4_, *aac(6’)-Il*, *aadA23*, *aadA2b*, *ant(2’’)-Ia*
pAMS-38b	109,015	50.8	144	ND	IncFIB(pHCM2)	ND
pAMS-38c	9525	51.7	15	ND	ND	ND
pAMS-38d	2511	51.8	2	ND	ND	ND

* ND: not detected.

**Table 2:** Features of *mcr-9*/IncHI2 plasmids similar to pAMS-38a.

	Plasmid	Distance To pAMS-38a *	Bacterial Species	Isolation Year **	Country	Accession No.
1	pC45-VIM4	0.0022	*E. cloacae complex*	2014	France	LT991958
2	pME-1a	0.0026	*E. hormaechei* subsp. *steigerwaltii*	2018	USA	CP041734
3	pCTXM9_020038	0.0048	*E. hormaechei*	2016	China	CP031724
4	pMRVIM0813	0.0049	*E. cloacae*	2015	USA	KP975077
5	pKPC-272	0.0079	*E. cloacae*	2012	USA	NZ_CP008825.1
6	p707804-NDM	0.0080	*Leclercia adecarboxylata*	ND	China	MH909331.1
7	pT5282-mphA	0.0108	*E. cloacae*	2012	China	KY270852
8	pN1863-HI2	0.0120	*E. cloacae*	ND	China	MF344583

* as detected by a Mash distance search against the PLSDB using the whole pAMS-38a sequence; ** ND: not determined.
